# Preventive healthcare policies in the US: solutions for disease management using Big Data Analytics

**DOI:** 10.1186/s40537-020-00315-8

**Published:** 2020-06-23

**Authors:** Feras A. Batarseh, Iya Ghassib, Deri (Sondor) Chong, Po-Hsuan Su

**Affiliations:** 1 College of Science, George Mason University, 4400 University Dr., Fairfax, VA 22030 USA; 2 School of Dentistry, University of Michigan, 1011 North University Ave, Ann Arbor, MI USA; 3 School of Engineering, George Mason University, 4400 University Dr., Fairfax, VA 22030 USA

**Keywords:** Big Data Analytics, Chronic diseases, Data incompleteness and imputation, Patients’ clustering, Preventive healthcare policy

## Abstract

Data-driven healthcare policy discussions are gaining traction after the Covid-19 outbreak and ahead of the 2020 US presidential elections. The US has a hybrid healthcare structure; it is a system that does not provide universal coverage, albeit few years ago enacted a mandate (Affordable Care Act-ACA) that provides coverage for the majority of Americans. The US has the highest health expenditure per capita of all western and developed countries; however, most Americans don’t tap into the benefits of preventive healthcare. It is estimated that only 8% of Americans undergo routine preventive screenings. On a national level, very few states (15 out of the 50) have above-average preventive healthcare metrics. In literature, many studies focus on the cure of diseases (research areas such as drug discovery and disease prediction); whilst a minority have examined data-driven preventive measures—a matter that Americans and policy makers ought to place at the forefront of national issues. In this work, we present solutions for preventive practices and policies through Machine Learning (ML) methods. ML is morally neutral, it depends on the data that train the models; in this work, we make the case that Big Data is an imperative paradigm for healthcare. We examine disparities in clinical data for US patients by developing correlation and imputation methods for data completeness. Non-conventional patterns are identified. The data lifecycle followed is methodical and deliberate; 1000+ clinical, demographical, and laboratory variables are collected from the Centers for Disease Control and Prevention (CDC). Multiple statistical models are deployed (Pearson correlations, Cramer’s V, MICE, and ANOVA). Other unsupervised ML models are also examined (K-modes and K-prototypes for clustering). Through the results presented in the paper, pointers to preventive chronic disease tests are presented, and the models are tested and evaluated.

## Introduction and motivation

**Fig. 1:**
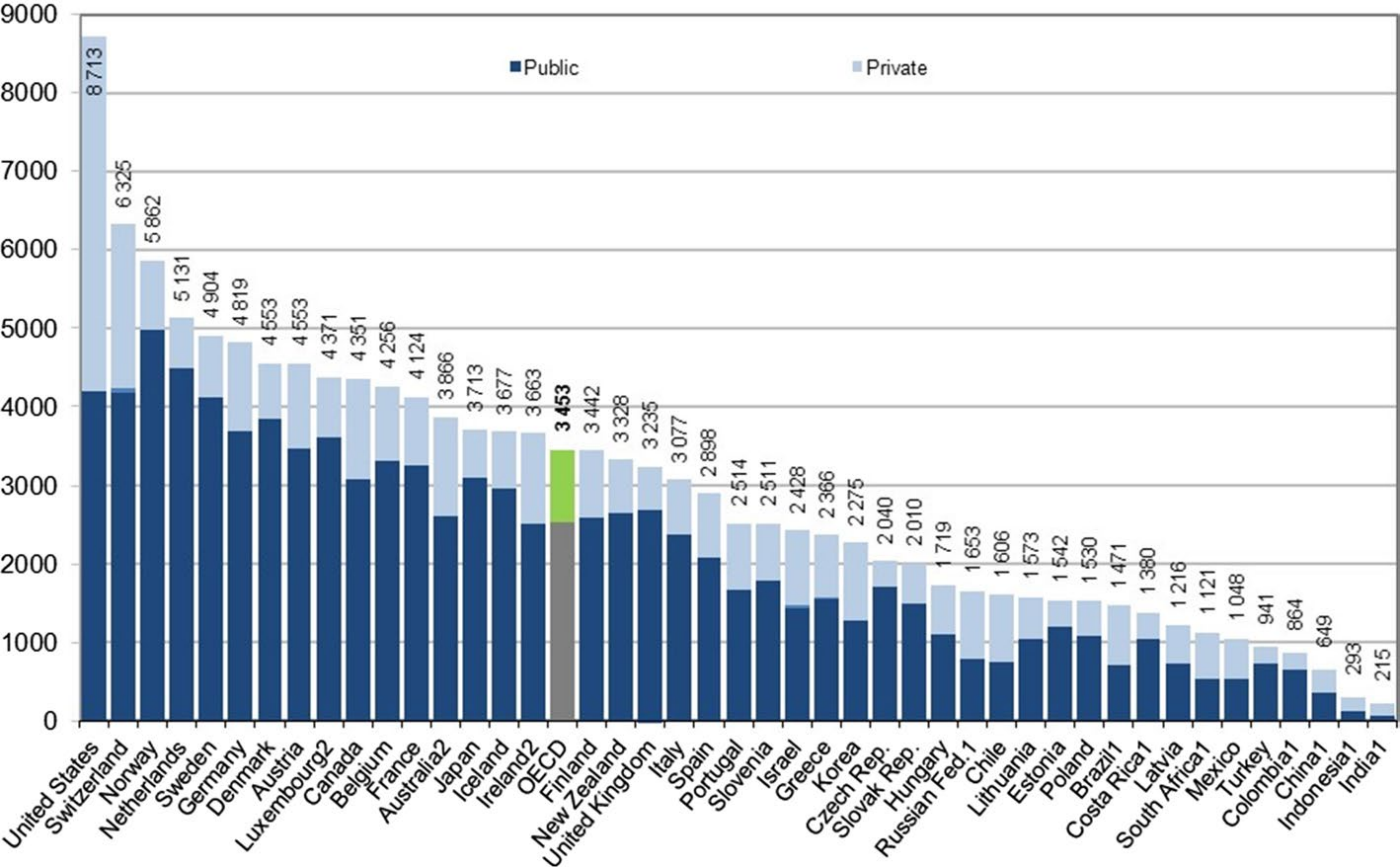
US healthcare expenditures vs. other OECD countries [6]

Prevention is one of the most important pillars of public health. The Institute of Medicine (IOM) estimates that *missed prevention opportunities* cost the US $55 billion every year, and an estimate of ~ 30 cents on every healthcare dollar. In total, the healthcare system squanders $750 billion a year [1]. Accordingly, amongst all the countries of the OECD (the Organization for Economic Co-operation and Development), the US has the highest expenditures (illustrated in Fig. 1). Preventive measures are economically critical. They are very clear pointers to the quality of service at hospitals and clinics; as well as the general health of citizens across any country.

Our work’s main objective (hypothesis) is two-tier: *through one of the largest and most representative national health datasets for population*-*based surveillance, data imputations and machine learning models (such as clustering) offer preventive care pointers by grouping patients into heterogeneous clusters, and providing data*-*driven predictions and policies for healthcare in the US.*

In our work, preventive care is measured and affected through three main parameters: 1-Immunizations, 2-Access to Healthcare Providers, and 3- Chronic Disease Prevention as indicated in United America’s Health Ranking [2]. We explain in detail these three important parameters in subsequent sections.

### Immunization policy

Immunization policies directly influence the health of a state. Different states in the US adopt different levels of immunization enforcement. Immunizations prevent the occurrence or spread of Vaccine Preventable Diseases (VPDs). For example, in the 20th century; the annual morbidity of smallpox was 48,164. Due to vaccines, in the year 2000 that number dropped to 0. Measles dropped from an annual morbidity rate of 503,282 to 81; and Rubella from 47,745 to 152 [3]. All VPDs had a reduction of more than 98% due to immunizations [4, 5].

**Fig. 2:**
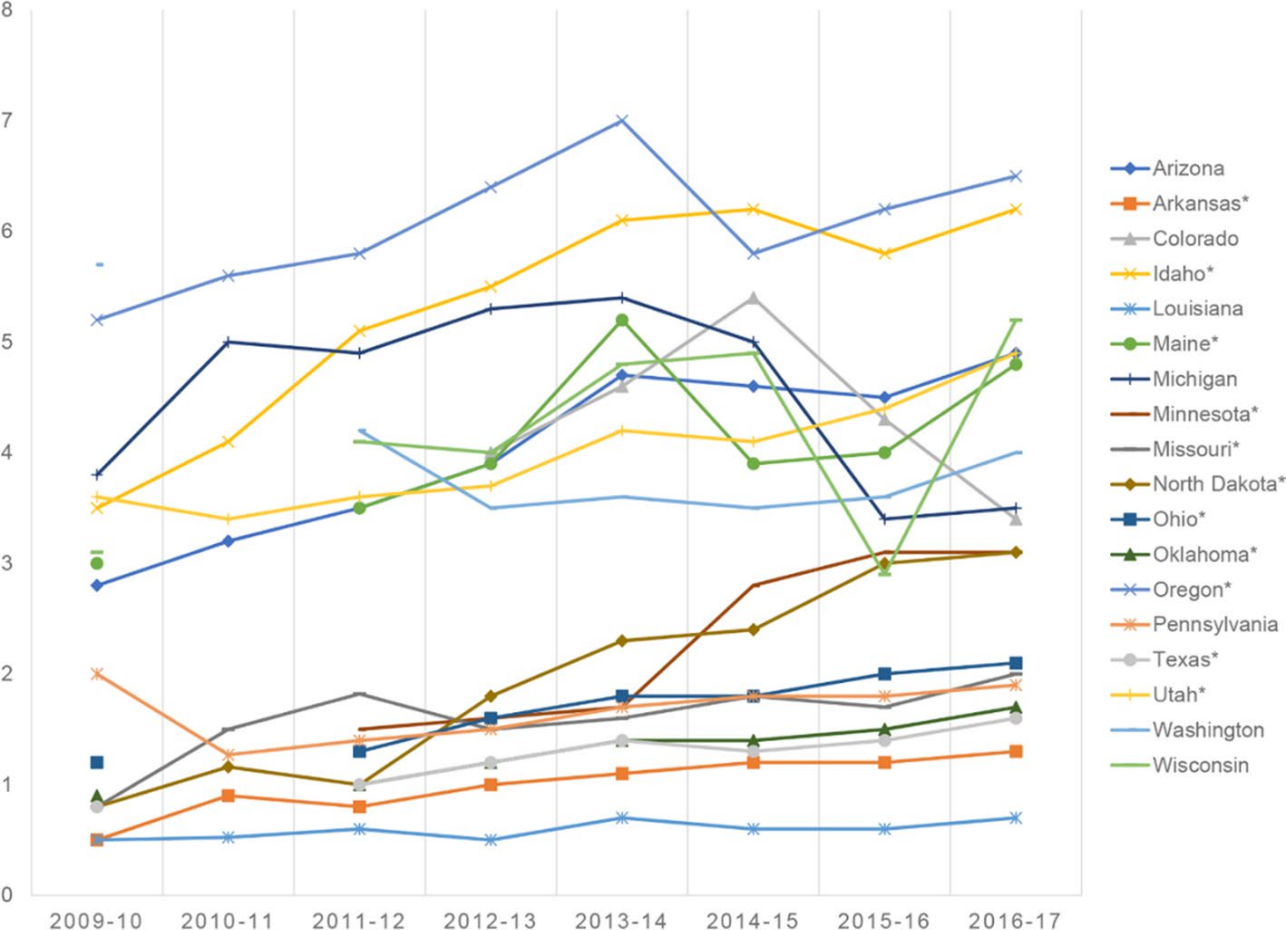
NMEs trends across the US [7]

Therefore, the case for immunizations has been considerably studied. States with low vaccine rates (such as Missouri, Indiana, Alaska, and Mississippi) hold campaigns to change public belief about immunizations. Due to the *lack of public trust* in vaccines and pharmaceutical companies, immunization rates have been declining. Figure 2 shows how Non-Medical Exemptions (NMEs) are on the rise in many states across the country (between years 2009 and 2017). The study declared the following conclusions [7]: “A social movement of public health vaccine opposition has been growing in the US in recent years; subsequently, measles outbreaks have also increased. Since 2009, the number of *philosophical*-*belief* vaccine NMEs has risen in 12 of the 18 states that currently allow this policy; namely: Arkansas (AR), Arizona (AZ), Idaho (ID), Maine (ME), Minnesota (MN), North Dakota (ND), Ohio (OH), Oklahoma (OK), Oregon (OR), Pennsylvania (PA), Texas (TX), and Utah (UT)”.

The Center for Disease Control and Prevention (CDC) reported on those states, and presented multiple cases to help increase public trust in immunizations: “We hope this report is a reminder to healthcare professionals to make a strong vaccine recommendation to their patients at every visit and make sure parents understand how important it is for their children to get all their recommended vaccinations on time” [5, 8]. Immunization rates vary among states, and lag behind the Department of Health and Human Services’ *Healthy People 2020* targets. It is yet to be determined how states will react to the recent outbreak of Covid-19 and its vaccination when that is made available.

### Access to healthcare

The second factor that affects preventable healthcare is access to healthcare providers. It has high correlation with *poverty rates*, *education levels*, and *job rates*; (i.e. it can be considered an economic attribute). Socioeconomic factors are heavily implicated with low or poorer management of disease and preclude patients from complying with seeking early screening and preventive care [9]. From a healthcare policy perspective, access is due to factors such as the lack of physicians in many areas of the country. In the US, in 2013, the ratio of *physician*-*to*-*patient* was *2.6*-*to*-*1000*, which is below the OECD recommended average of *3.3*-*to*-*1000* [8]. Additionally, OECD market research predictions indicate that by the year 2025, 52,000 more primary care physicians are needed to meet demand; especially due to the ACA’s provisions to increase that number [10]. While the US average for doctors active in patient-care per 100,000 people = 225.6 (in 2014), there is a *wide* variance across states: Massachusetts ranks the highest with 349.5 active doctors per 100,000 people (the ACA is a reincarnation of the Massachusetts healthcare system). Mississippi has only 170.3 doctors per 100,000 people [10, 11].

**Fig. 3:**
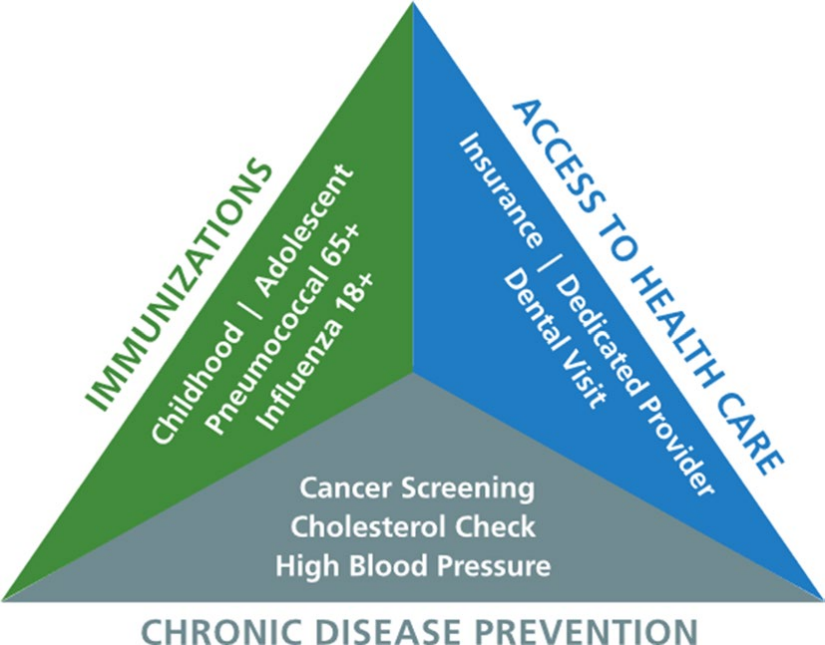
The three pillars of preventive healthcare [2]

In this manuscript, we present methods that focus on the third (and most critical) variable in preventable healthcare: Chronic Disease Prevention and Prediction (CDPP) (Fig. 3).

Most chronic diseases are linked to risk factors or indicators such as obesity, smoking and inflammatory conditions. As a result, sub-clinical changes are potentially occurring with subjects, and that can be detected prior to clinical manifestations. In addition to indispensable clinical tests and screening, a programmable bio-nano-chip (PBNC) system for example could be used in enabling the detection of proteins and molecules known as biomarkers that facilitate ‘*early*’ diagnosis for possible disease prevention. This technology is generally referred to as a *lab on a chip (L*-*O*-*C)*. One example is the blood glucose monitoring device. Thus, evaluation of changes occurring prior to disease appearance (preventive) may highly lead to better management of the disease and is likely considered more cost-effective. When physicians are presented with such projections, they can augment their recommendations to patients. Patterns can be drawn between patients’ clinical and laboratory data, passed to predictive and intelligent models for proactive actions. These attributes are collected in this study using CDC data, and pointers to enforcing CDPP using ML are presented.

### Chronic Disease Prevention and Prediction

Chronic diseases encompass diabetes mellitus (type II), hypertension, kidney diseases, obesity, inflammatory diseases, and cancer, among others. Nearly half (45%) of Americans have at least one chronic condition [12]. Direct medical costs for chronic conditions are > $750 billion annually. Further, by 2023, CD cases will increase by 42%, costing $4.2 trillion in treatment. Choosing the right combination of diagnostic tests requires the understanding of the disease, and managing it [13].

The list of tests provided through the ACA is designed to help eradicate chronic diseases [14]. Out of the four possible healthcare models for a country (*The Beveridge, Bismarck, Out of Pocket, and National Insurance*) [15], a model such as the ACA provides guarantees for the pursuit of preventive healthcare. However, in recent years, the ACA has been deployed with high costs, the program has low adoption rates, and it still suffers from public’s rejection. Within the ACA, all marketplace plans must cover the following list of 21 *preventive* services without charging the citizen a co-payment; those are presented in Appendix 1 (verbatim from Healthcare.gov) [14].

Data on the 21 listed biomarkers are collected by CDC. One of the major sources of such CDC data is their National Health and Nutrition Examination Survey (NHANES) [16] (used in this work). NHANES is a program that is concerned with population-based surveillance of health and disease via surveying; that refers to patients’ reported outcomes, collecting laboratory and clinical data, as well as demographic and socioeconomic statuses of the US population. Datasets are released in 2-year cycles. The program is part of the National Center for Health Statistics; one of the thirteen US government statistical agencies. Policy improvements through CDC big data and the ML methods applied make a strong case to enforcing CDPP best practices in the clinic and on a national scale.

Our study provides completeness to the collection of biomarkers through learning from clinical historical trends. Our ML models can improve preventive practices at clinics, save doctors and patients time, reduce cost of unnecessary clinical tests, and provide improved diagnostics to incoming patients by essentially categorizing them in health and disease groups using k-clustering.

## Data and healthcare—a literature review

In the world of silicon chips, Moore’s Law is still alive and well [17]. The law suggested the doubling of speed and capability of computer chips every 24 months; which still holds true. Interestingly though, the same logical construct has been applied to other areas. Eroom’s Law (*reverse of Moore*) suggested that the cost of developing new drugs has doubled every nine years since 1950 [18]. That alludes to more drugs being used for cure, but less towards prevention. This section presents the one of the largest US public health dataset that can aid in preventive practices (i.e. NHANES), and presents a history of ML for healthcare.

### CDC data for preventive healthcare

In 2007, the Bush administration presented the Food and Drug Administration Amendments Act [19]. The act reaffirmed the requirement for federal investigators to release partial information about clinical trials. Before the act, government agencies had no incentive to collect clinical data, or release comprehensive datasets to the public for research and policy validations.

Later on, when the Obama administration enacted the Open Data Initiative [20]; all government agencies became obliged to share their public data through online repositories.

The CDC (through NHANES) has been performing health surveys since 1956. They cover hundreds of health attributes for a patient at a single point in time [21]. Clinical trials and surveying are critical sources of information for such medical innovations. Time series data (*longitudinal*) are important for ML models. Predictions, forecasts, and other means of pattern recognition are strictly enforced by a time dimension in the data. NHANES data are cross-sectional (i.e. collected at a single point in time); thus a follow up on patients is required for ML trends and forecasts [16]. Completeness of health data is required to allow for comprehensive examinations and to provide early recommendations to preventive parameters. For example, an increase in blood pressure through time can point to cases of hypertension in some patients. In this work, we examine the nature of NHANES data, allocate imbalance and incompleteness, and solve that using correlations, clustering, and imputation methods.

### Machine Learning for healthcare

Since the early inception of Artificial Intelligence (AI), healthcare has been at the forefront of applications. One of the first forms of AI is expert systems. Knowledge-based systems (KBS or expert systems) are intelligent systems that encapsulate the knowledge of a skillful person. KBS are a special kind of an intelligent system that makes extensive use of *knowledge*. KBS were first introduced in the 1960s during the process of collecting medical knowledge from healthcare practitioners. KBS are different from conventional software systems or data analytical systems because they use *heuristic* rather than *algorithmic* approaches for decision making [22].

The original idea of a general problem solver (GPS), that later turned into the idea of building a medical expert system, used generic search techniques aided by heuristic knowledge to solve medical problems [23]. The GPS idea was instrumental in the development of MYCIN; a system that diagnosed blood and hemoglobin disorders. MYCIN is a *landmark* medical KBS developed at Stanford University (known to be the first expert system). PROFORMA was later developed as a generic model for building clinical and medical expert systems (due to the rise in demand for such systems) [24]. Data re-kindled the promise of AI and fueled the algorithms that struggled to provide learning and intelligence due to the lack of abundant datasets [25]. That led to the transformation of the famous adage “*data is the new oil*” to “*data is the new blood*”.

**Fig. 4:**
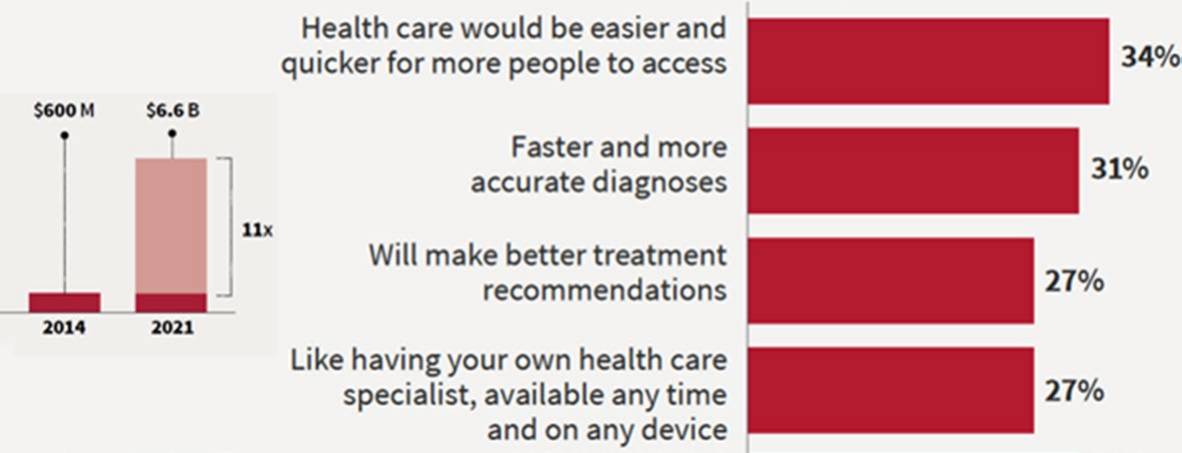
Growth of AI in healthcare [26]

In 2018, at Stanford University (the developer of MYCIN), healthcare and ML are hand-in-hand at the Center for Clinical Excellence (CERC). In their recent publication [26], they point to multiple endeavors at their centers to deploy AI/ML in healthcare. Additionally, they point to the rising trend of AI in the healthcare domain, and how it is expected to grow (percentage-wise and 11-fold) by year 2021 (Fig. 4). Many medical research labs and clinics are starting to deploy ML models within their practices, albeit still considered scarce [25, 27].

### The data replication crisis

Before presenting our models, it is very important to point to a very critical and dangerous phenomenon in healthcare research and deployment of data-driven methods: *the replication crisis*; something we avoid in our study by using open data, and providing our models, code, and datasets to anyone interested in replicating our study.

Data for scientific research need to be reliable. “Reliable” in this context means the following: 1-unbiased to one geographical location, 2-unbiased to one or few demographics, 3-unbiased to certain arguments for a policy by candidates on the political left or right 4—data modeling needs to avoid issues such as overfitting/underfitting or imbalance. Although the majority of clinical studies are dependent on data collected at one clinic, hospital, or university, there has been major pushback on medical studies and the lack of ability to regenerate the results with a different set of patients, pre-conceived clinical setup, a variety of geographical locations, inherited biases in the analysis, or hyper-parameter’s tuning that is specific to the results of one study—all those practices lead to an inability in replicating a study [28–30].

NHANES is an open and democratized dataset that represents national health and disease. It is also claimed to be verified by multiple practitioners throughout the years. Like other big data repositories, albeit being incomplete, NHANES was used for our models. We allocated biases or inconsistencies, solved these issues, and *then* built our models.

The remaining of the manuscript is organized as follows: the next section presents the CDC big data management processes using SQL, bias removal, and data wrangling. “Results: preventive healthcare through Data Analytics” section presents the ML models (clustering, correlations, and imputations), and their results (result #1–result #7). Lastly, “Discussion: conclusions, policies, and future plans” section demonstrates conclusions and implications on preventive policies.

## Methods: CDC data collection and bias detection

Data are collected through the CDC’s NHANES web-pages. Files get extracted into a SAS viewer, and then exported into a SQL database management studio. All the tables had one *primary key:* SEQN, which represents a *distinct* sequence ID for every patient. The database has 27 tables, a total of 1000 + data columns, and tens of thousands of data points (detailed later in this section). No other similar relational database that includes the majority of the CDC survey data exists in literature.

### Preventive health variables from the CDC

Most years within the NHANES surveys have missing data. Righteously however, the CDC published a report declaring the reasons, counts, and summaries of missing data from their surveys. In the report, the following reasons are mentioned (verbatim): “the answers are classified as unusable; the respondent does not have the information to answer a particular item or refuses to answer a specific question or undergo a particular test; laboratory equipment fails; test results are faulty; specimens are lost in shipping; or some items of information fail to be recorded on the examination record”.

Reasons, as such, were only documented after 1988. With data collected previous to that year, it is unclear what pitfalls exist (the *electronic collection* survey began in 1971). NHANE’s history of data collection is broken into the following five phases:

1. 1971–75—National Health and Nutrition Examination Survey I (NHANES I)
2. 1976–80—National Health and Nutrition Examination Survey II (NHANES II)
3. 1982–84—Hispanic Health and Nutrition Examination Survey (HHANES)
4. 1988–94—National Health and Nutrition Examination Survey (NHANES III)
5. 1999–present—National Health and Nutrition Examination Survey (Continuous NHANES)

In this study, we investigate data from 2009 to whatever is available up to 2019—an effort to cover the last 10 years of surveying. It is worthy to mention that every batch of NHANES has a different set of patients (and consequently a different set of patient IDs).

As one can assume, the sample citizen set which NHANES presents is not biased or imbalanced to any demographical group (we challenge that in upcoming sections). NHANES claims that they sample the data in accordance with percentages by the US Census Bureau; they consider Race, Age, Income, and Gender as basis to stratify the population. In their guides, NHANES claims the following: “Race and Hispanic-origin information used for sampling is based on census population estimates and obtained from the household screener to determine eligibility for inclusion in the survey” [16]. One can argue that stratification is not of relevance to a patients’ medical status, and in order to cover a wider part of the variety in the clinical spectrum, stratifying needs to *be performed based on medical variables*. Most importantly however, the data are not found to be longitudinal; it is therefore not possible to completely follow a patient’s progress over time. Patients are presented in ID forms, but multiple variables on their demographics, habits, medical history, clinical tests, and multiple other important variables are recorded. We collected data from all six groups (Demographics, Dietary, Examination, Laboratory, Questionnaire, and Limited Access). Out of these six groups, we traced 27 preventive care categories. Those are:

*Questionnaires Group:* 1-Cholesterol, 2-Cardiovascular health, 3-Current health status, 4-Diabetes, 5-Diet behavior, 6-Drug use, 7-Hospital Utilization and Access to Care, 8-Medical Conditions, 9-Oral health, 10-Four parameters of smokers, 11-Weight history, 12-Smoking—Cigarette, Use 13-Smoking—Household smokers, 14-Smoking—Recent Tobacco Use, 15-Smoking—Secondhand Smoke Exposure. *Demographics Group:* 16-Variables and Sample Weights. *Examination Group:* 17-Blood Pressure, 18-Oral Health—Dentition. *Laboratory Group:* 19-Standard biochemistry profile 20-Plasma fasting glucose 21-Insulin 22-Glycohemoglobin 23-Feritin 24-Cholesterol-Total 25-Cholesterol-LDL 26-Cholesterol-HDL 27-Complete Blood Count with 5-part [16].

The mentioned 27 diagnostic categories include 1000 + variables, all of which are considered in the ML models (clustering and imputations). Prior to developing the models, a significant effort of data wrangling is deployed, that process is presented next.

### Experimental setup and data wrangling

After data collection and transformation, all tables have been merged into one database view within the *dbo schema*; analysis has been performed on the unified table. For example, a SQL code for merging Cholesterol levels data with smoking, cardiovascular health, and diabetes is as follows (*Inner joining tables on SEQN*):
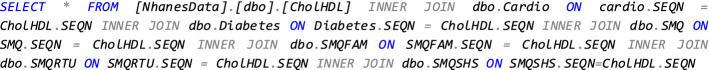


When the data are merged, several observations are possible. These observations span the entirety of the NHANES data. Observations (presented as *result #1* of this manuscript) on the merged, cleaned, and wrangled data are:

1. Data categories did not have the same counts, which could lead to data imbalance and lack of comprehensive experimentation in terms of preventive parameters.
2. Demographic data are the largest. The dataset included more than 40,000 patients (Fig. 5 summarizes counts of all NHANES variables).

**Fig. 5:**
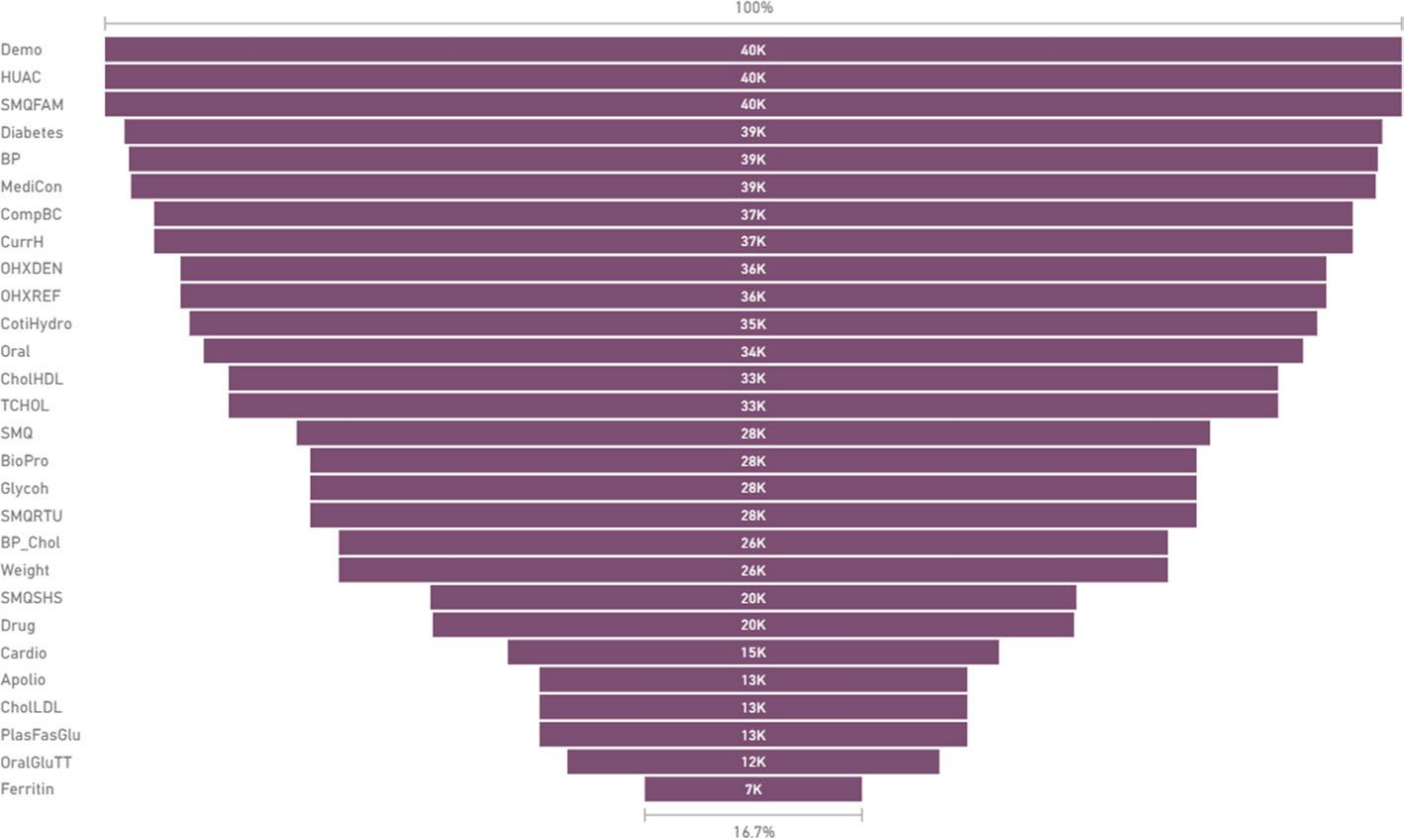
Counts of patients per category in NHANES data for 2009 onwards
3. Demographical categories are not equal, not balanced, and had a serious case of bias towards certain demographic factors (Figs. 6, 7, 8, and 9 show the demographic categories and their imbalance—something that could be improved within CDC’s data collection process).

**Fig. 6:**
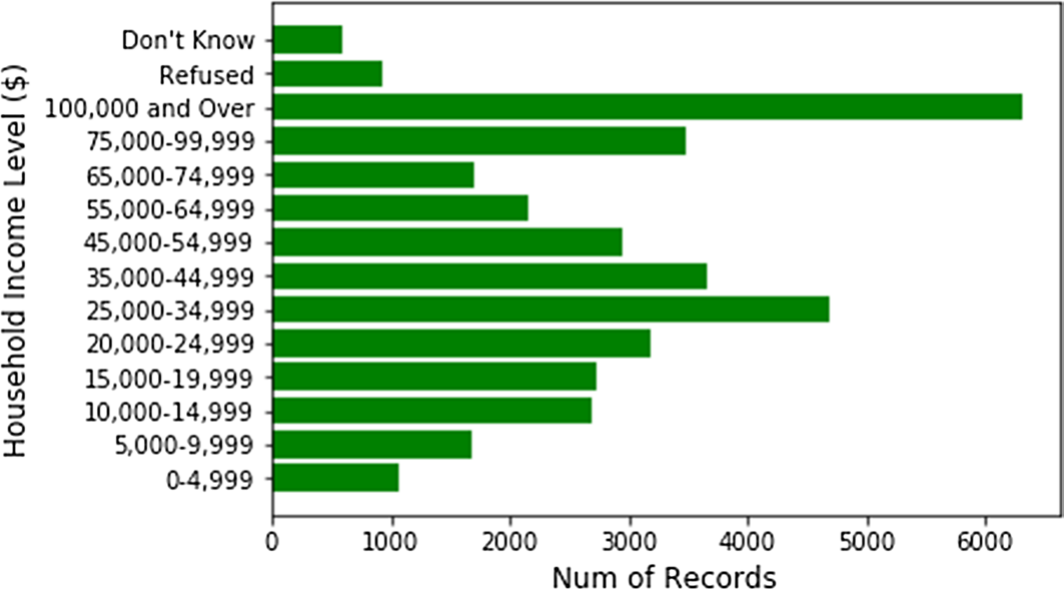
Household income counts (solely from the household table)

**Fig. 7:**
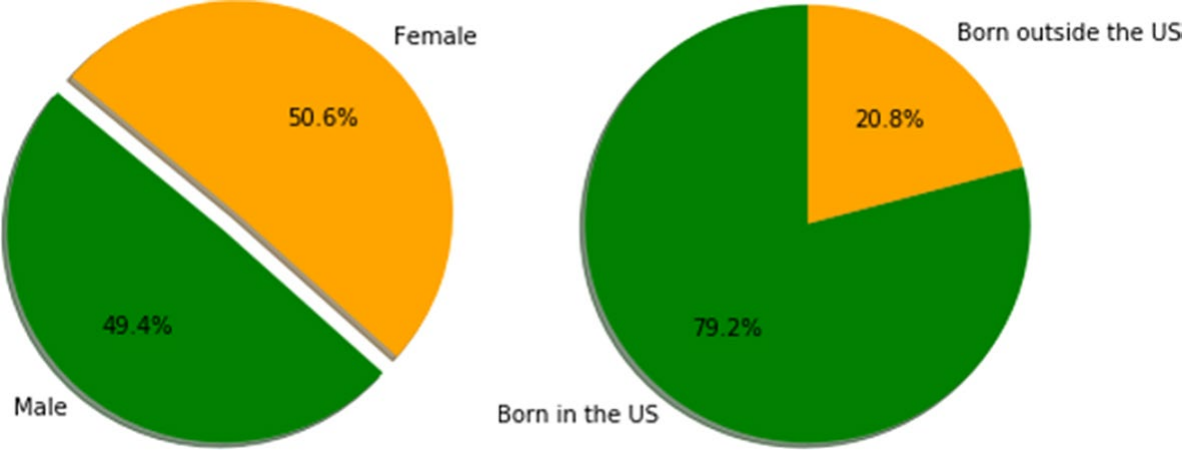
Gender and birth percentages in CDC surveys

**Fig. 8:**
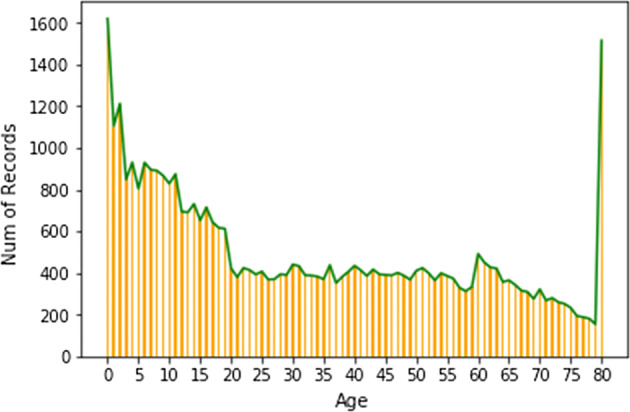
Age counts in CDC surveys

**Fig. 9:**
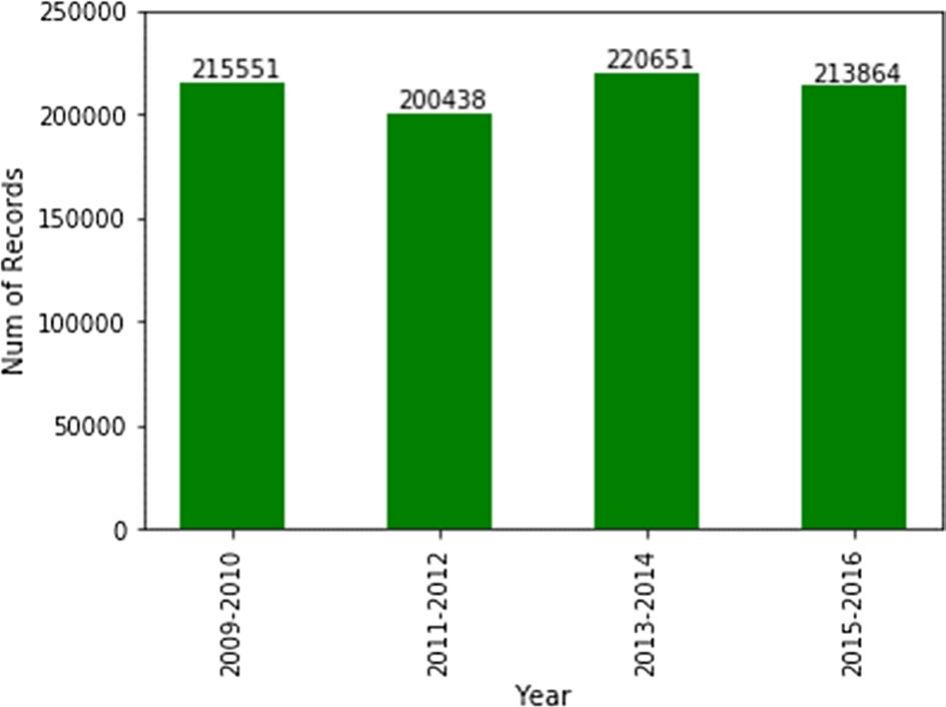
Number of patients by NHANES survey year
4. Ferritin data (for instance) did not have any overlap in terms of patient IDs with any other category.
5. The majority of surveyed patients’ household income is > $100,000 (i.e. more well-off patients are surveyed in comparison to middle class & poor patients).
6. When merged on patient IDs, the data size reduces drastically, due to the minimal overlap between different categories (for clustering, we ended up clustering more than resulted ~ 1500 patients, instead, we used the imputed data as well).
7. Female vs. male distributions are fairly unbiased. However, when it comes to race, some races, such as Hispanics, are not well represented in the survey (Fig. 10). Studies show that Hispanics, along with African Americans have higher level of diabetes than other races [31]. Therefore, for preventive parameters of diabetes, data for more races ought to be collected or created.

**Fig. 10:**
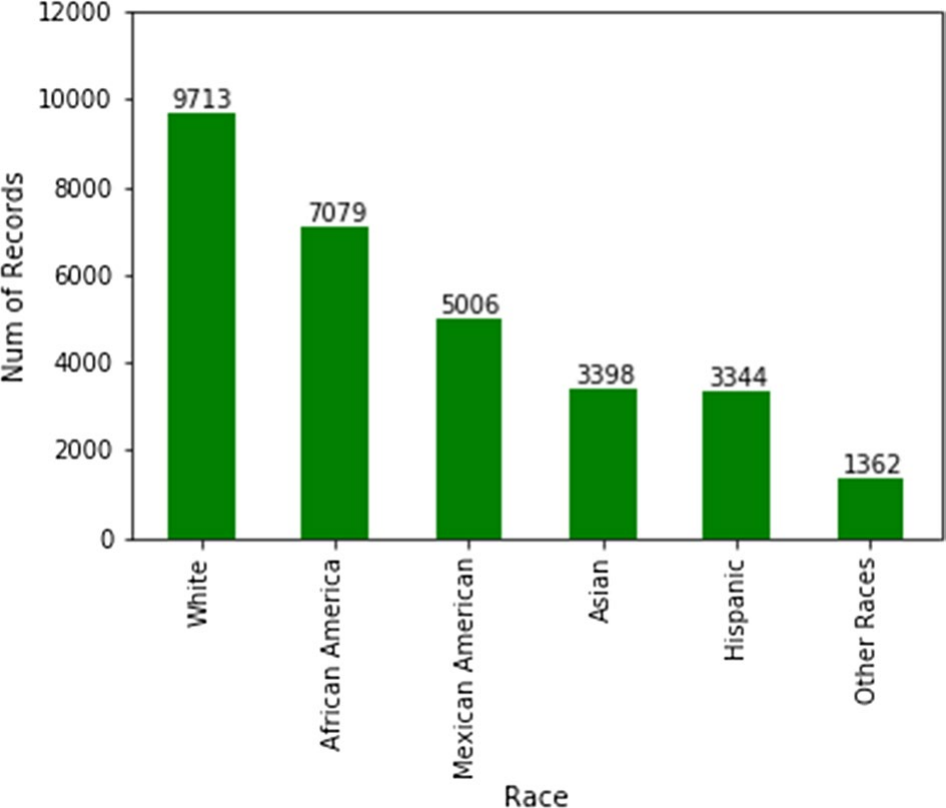
Counts of patients per race in NHANES data

The NHANES data collection process was rather costly. Therefore, as an attempt to substitute for the need to acquire expensive utilities needed to collect clinical parameters, data imputation methods were applied to compensate for missing clinical data. These methods are applied in  “Multivariate imputation by chained equation” to NHANES 2015-2019 datasets with missing clinical oral health parameters and smoking variables as examples.

Table 1 summarizes all the data categories’ counts across the NHANES survey database (*presented as result # 2*). After cleaning, wrangling and merging the data, the method presented in the next section could be deployed to solve any NHANES data imbalance issue. The results introduced in  “Results: Preventive Healthcare through Data Analytics” section are presented through multiple dimensions such as *dental diseases*, and *smoking (SMQ)*.

**Table 1:**
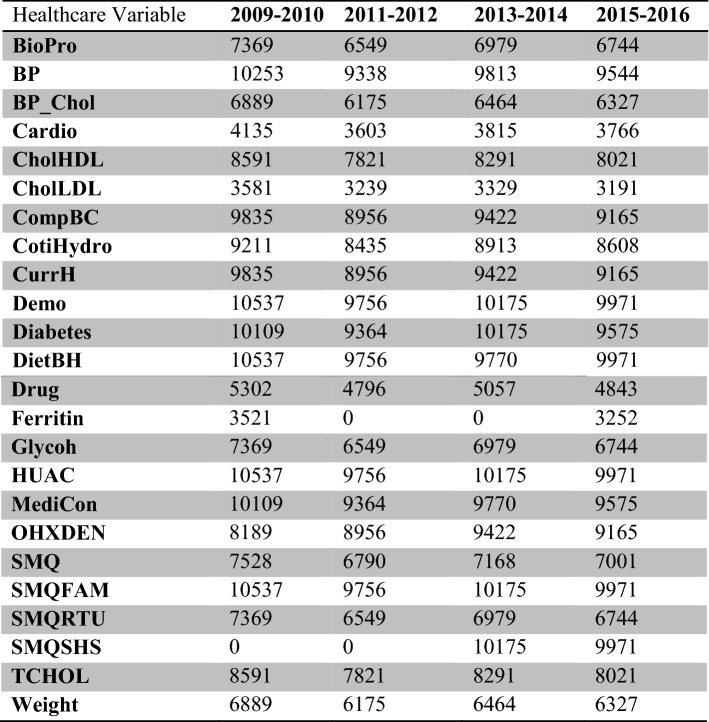
Summary of data for preventive parameters in NHANES

The next section presents the experimental methods used for imputations of preventive biomarkers (in accordance to what was proposed by the ACA), and the ML models deployed through the database.

## Results: preventive healthcare through Data Analytics

This section introduces the models and the results for data imputations, as well as the clustering model for patients included in the CDC surveys. The next two sub-sections include the following: 1—Imputations using correlations (Pearson and others) for data creation of variables that can point to preventable diseases 2—A *multivariate* clustering model of patients, based on all variables mentioned above. The goal of the model is to group patients into multiple clusters for preventive recommendations and management.

### Multivariate imputation by chained equation

The data wrangling process is referred to as *80% of the effort* in a data science lifecycle. The most challenging aspect in data wrangling is data incompleteness. Incompleteness leads to data imbalance, and therefore, leads to biased outputs from models. Incomplete data often makes the case for a Garbage-in Garbage-out (GIGO) situation; therefore, imputation is deployed. Most commonplace methods for imputation are presented below. None of the four mentioned methods are useful in the case of CDPP—the reasoning is presented in parenthesis:

1. Providing means, modes, and medians of a column to *replace* missing data (Averages of patients do not mean anything—patients respond to diseases in unique fashions). It is noteworthy to state that since NHANES data are cross-sectional, stratification is not possible. Such imputations are applied when monitoring patients longitudinally.
2. Providing means, modes, and medians of data points surrounding missing data—usually 3 on each side, or more (Same reason as in #1. Closer patients in order are not closer *health*-*wise*).
3. Using correlations with few predefined columns to extract new values (Mostly used in small datasets. The number of columns that are used in this study is ~ 1000+, which makes it impossible to find one or few columns to be used for correlations; instead all columns ought to be used).
4. Deleting rows with missing data (Deleting rows/patients will lead to bias problems, and so we avoid this option).

Instead, we introduce a different process for data imputation of CDPP variables. The method used includes *six* main steps:

1. Stream data from the *SQL* database into the R environment, split data into testing and learning data, use learning data for all steps except step # 6.
2. Measure correlations between every column and all other columns to find the highest correlated columns for every CDPP variable.
3. Apply Pearson correlations for *numerical* values, Analysis of Variance (ANOVA) for columns that have *numerical and categorical* values, and Cramer V correlation coefficients for data that are categorical:
  i. Cramer V coefficient is used when both *x* and *y* are nominal. Results are decimal values between 0 and 1. Formula for Cramer V is: *Φ *= SQRT (X^2^/N (K-1)). *ϕ* denotes Cramer V; *X*2 is the Pearson Chi square statistic; *N* is the sample size involved in the test; *K* is the lesser number of categories of either variable.
  ii. ANOVA is used when *x* is numeric, and when *y* is nominal (or vice versa). The result is a decimal value between 0 and 1.
4. Pass the top *10%* correlations for every column as inputs for the Multivariate Imputation by Chained Equation (MICE) model—an R library [32]. The MICE model then uses columns that have a correlation >* 0.5*.
5. Run through all columns of missing CDPP data and impute data points based on the highest correlated columns and MICE.
6. Validate created CDPP data versus actual data (using the testing datasets from Step #1).

A sample code from the MICE algorithm used for this study is as follows (used in Steps #4 and #5):
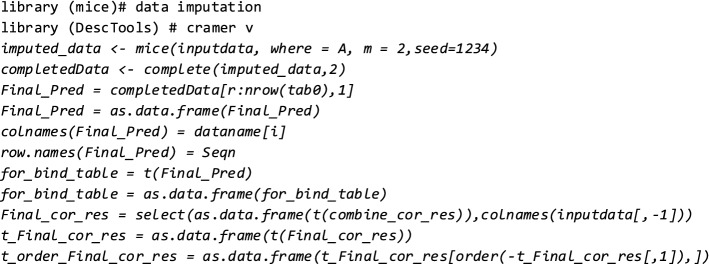


The 6-step process is applied to providing missing longitudinal smoking data as an example. Smoking is known to be one of the most common risk factors to many chronic diseases, therefore, completeness in this data is critical to providing health recommendations. The following successful results are produced:

1. Imputations error rate is: **0.071**. That is deemed to be very low (*result #3*).
2. The process created similar statistical distributions of predicted data and actual data.
3. Top correlations for disease and smoking data are identified (Fig. 11), with low error rates. Such variables aid medical teams in identifying healthcare parameters for preventive healthcare amongst a subgroup of the population taking the NHANES survey. This breaks down SMQ to specific practices and can aid in making a quick change. For example, *cigarette filter type* seems to have a high effect on the patient’s smoking numbers (correlation = 77%). *Cigarettes length* and *Tar content* are other two high effect variables—(*result #4*).

**Fig. 11:**
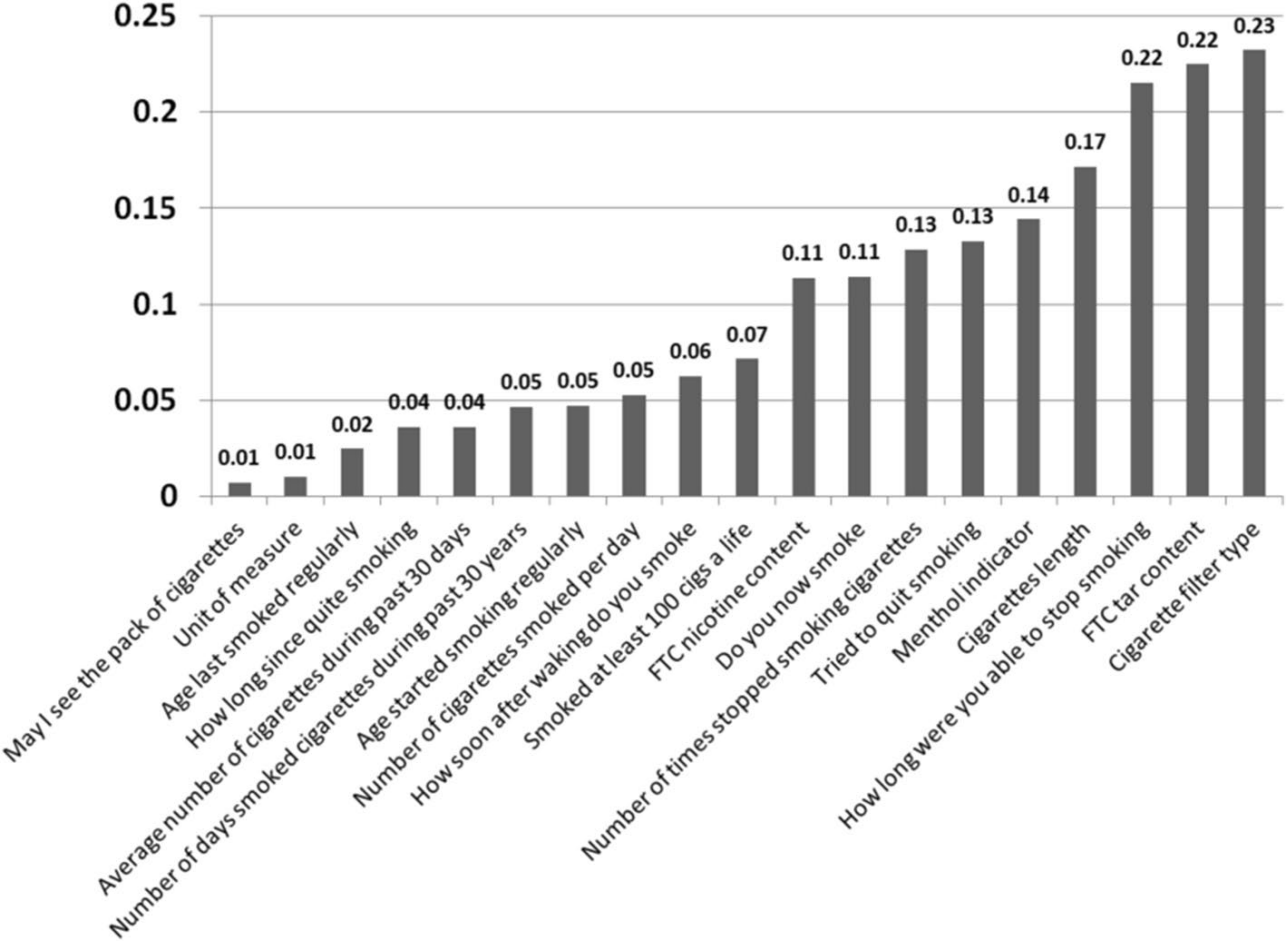
Error rates in correlations for top imputations of SMQ data variables
4. The listed measures (survey questions) are the preventive pointers to smokers—in order to avoid certain smoking-relevant diseases.

**Fig. 12:**
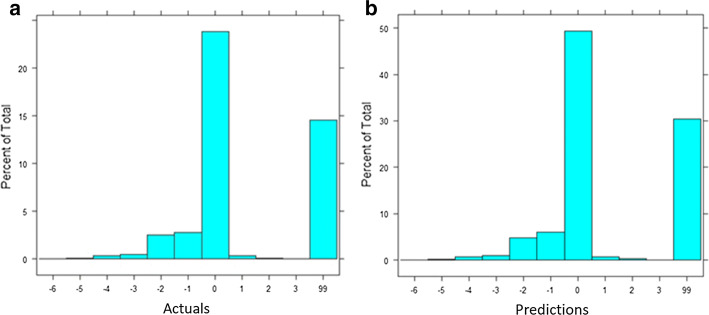
**a** Actual and **b** imputed/predicted periodontitis columns’ distributions are very similar

The 6-step imputations’ process of CD data can provide more detailed pointers to preventive sub-variables (such as the cigarette filter example). Another example presented is for missing clinical periodontal measures used to estimate prevalence of *Periodontitis*. Periodontitis is a host-inflammatory oral disease characterized by lengthy exposure to pathogens. For periodontitis, imputations created similar distributions of groups (actual data vs. predictions) amongst periodontitis predictions. See Fig. 12 for a visual comparison.

**Fig. 13:**
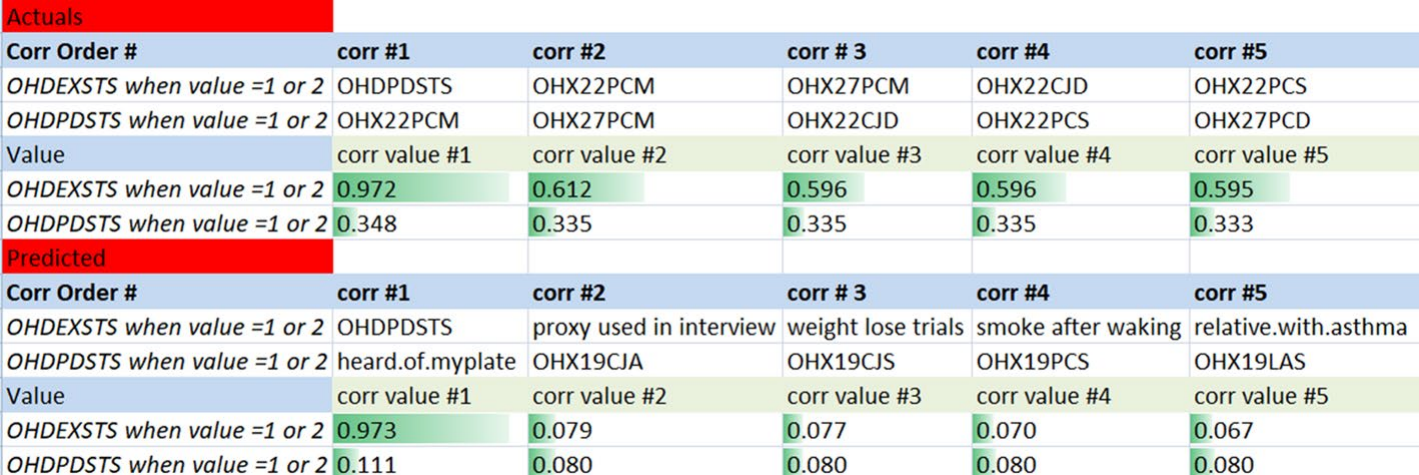
Correlations results (actual vs. imputation results, filtered by periodontal test)

Periodontitis of mild, moderate or severe form affects over 64 million Americans today; i.e. above 42% of the US population aged 30–79 [33]. This disease is heavily diagnosed and monitored via clinical parameters; thus, in case where there are limited resources or if dubbed costly or inaccessible, imputations can potentially serve to primarily identify patients requiring further screening, preventive or interventional measures. Periodontitis can be prevented and managed; therefore, a data-driven approach can present a state-of-the-art ML method to apply on a population; i.e. a large scale of individuals. Here, this was implemented on NHANES missing periodontal data for 2015 onward. When comparing actual vs. predicted results of imputations for periodontitis, and comparing the highest correlated variables between both sets, many variables ended up having similar correlations such as: *OHDPDSTS* dentition status, *OHXCJID* dentition status, and *OXHXPCM* dentition status. Figure 13 shows the top 5 variables that have the highest correlation.

**Fig. 14:**
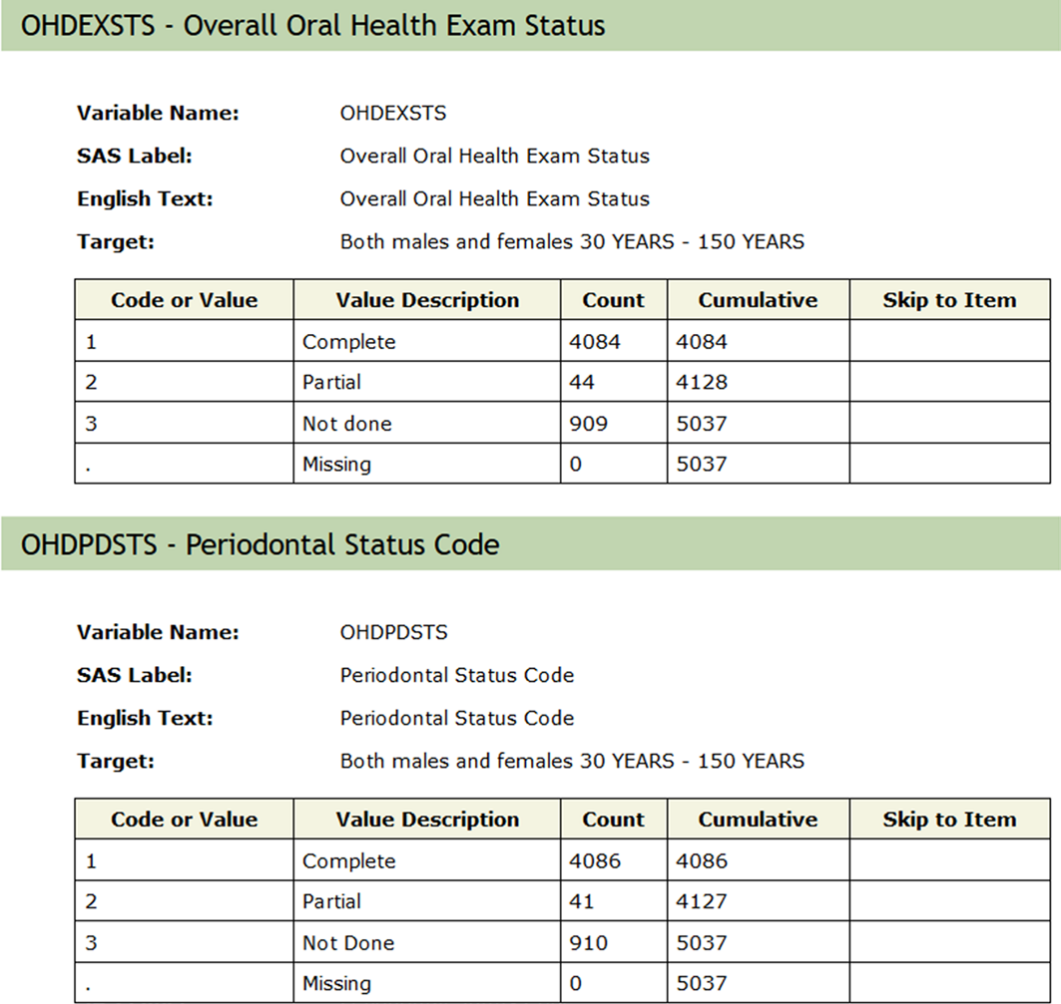
Example NHANES codes for patients that performed the periodontitis tests [16]

The dental variables’ filters used in the study are illustrated in Fig. 14 (showing the criteria for data collection of periodontal data in NHANES). Only the *complete* and *partial* dental tests are included, any data that are *not done* or *missing* are not considered in the imputations or correlations test.

Refer to Corr Order # for instance; it has filters 1 and 2. Corr#1 in actual data is the highest correlation variable (**0.972**) and corr#2 is the second highest (**0.612**), and so on. Similar to that, in predicted values, corr#1 is equal to 0.973, and corr#2 is equal to 0.079 (*result #5*).

The goals desired from the mentioned periodontitis example are to present initial pointers to the disease, and to point to factors that would allow for preventive dental parameters (providing focused dental cleaning and cures). The 6-step imputations model presented in this section provides completeness and balance to the datasets, however, to fit patients into a group of similar patients, a clustering model is required, that is presented next.

### Clustering patients

The k-modes clustering algorithm is an extension of the infamous k-means clustering model. Instead of distances, the k-modes model is based on *dissimilarities* (that is, quantification of the total mismatches between two data points: the smaller this number, the more similar the two objects). K-prototype (k is the number of clusters) [34] is used for clustering numerical and categorical values. It is a simple combination of k-means and k-modes. K-prototype has the following steps:

1. Select k initial prototypes from the dataset *X.*
2. Choose the number of clusters (Fig. 15 illustrates the **Elbow** diagram for recommendations of *k*).

**Fig. 15:**
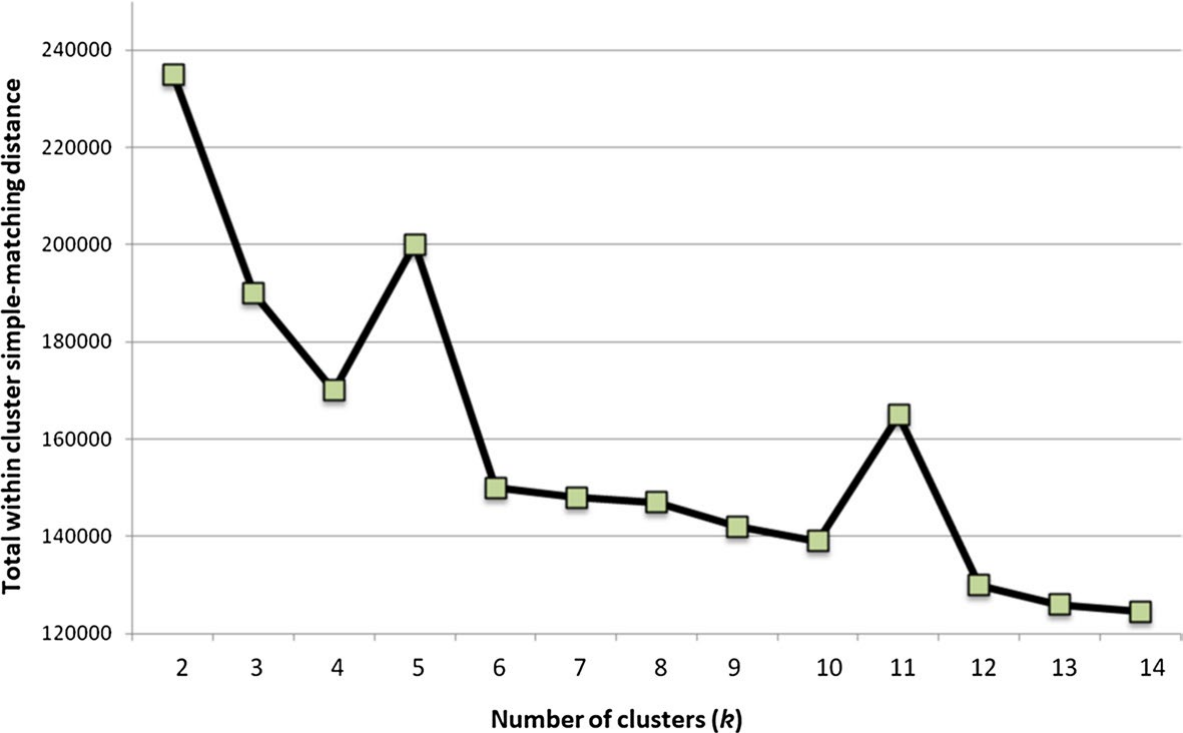
Diagram for choosing the number of clusters (k = 5 and k = 11 peaks)
3. Allocate each data point in *X* to a cluster whose prototype is the nearest.
4. Retest the similarity of objects against the current prototypes. If the algorithm finds that an object is allocated such that it is nearest to another cluster prototype, it updates the prototypes of clusters.
5. Repeat step #3, until no object changes its cluster (after fully testing *X*).

A variety of clusters’ descriptions could be pulled from the model developed. The model includes 519 variables; we only used columns that had *more than 10% values, and less than 90% nulls*. Columns with Null values could lead to model skew. Depending on the application deployed, the clusters could be defined depending on the purpose. For example, they can be defined based on smoking habits and blood pressure, but they will be categorized differently if they are defined by chronic diseases—the seven CDs collected in our results are (a total of 1711 cases):

1. Hypertension (477 cases)
2. Diabetes (188 cases)
3. Arthritis (373 cases)
4. Cancer (111 cases)
5. Asthma (173 cases)
6. Coronary Disease (41 cases)
7. Periodontitis (348 cases)

**Fig. 16:**
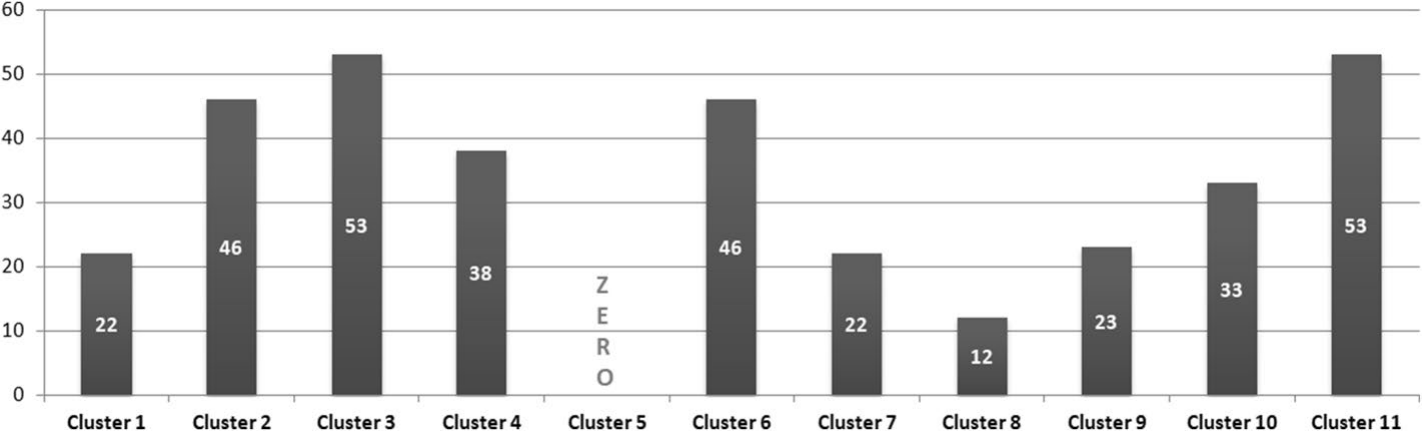
Patients with severe periodontitis within the 11 clusters (count = 348)

The best k-model has 11 clusters (based on the elbow method and clinical heterogeneity). For instance, it is worth mentioning that cluster 5 is the **‘healthy cluster’**. Cluster 8 has patients who don’t have high levels of Cholesterol (Mmol/L)—6 or higher. Clusters 3 and 11 are the ones with the highest probability of oral health disease (i.e. periodontitis); a 300 + sample of patients had periodontitis. As one can notice, clusters 3, 7, and 11 are the least healthy, while 5, 6, and 8 are the healthiest ones. Figure 16 presents the distribution of clustering results by periodontitis projections. More importantly, Table 2 presents the counts of CD patients within every cluster (*result #6*).

**Table 2:**
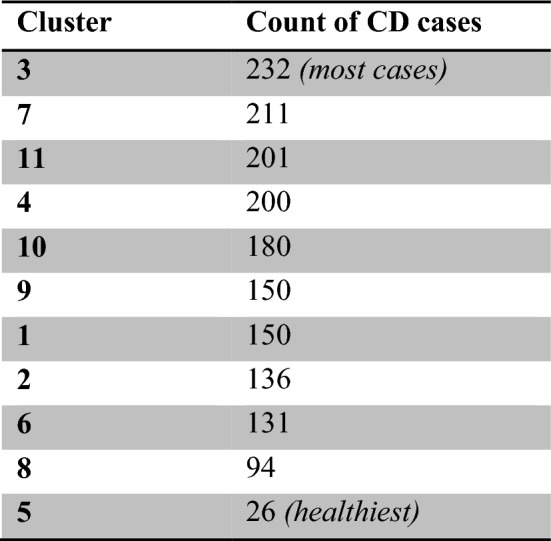
Summary of CD counts within every cluster (descending order)

Most chronic pro-inflammatory conditions have common risk factors, such as smoking and co-existence with other systemic diseases. In case of periodontitis, diabetes mellitus II is a chief risk factor to exacerbation of the disease. Both diseases were claimed to even have a bi-directional or a two-way relationship. Periodontitis is the 6th most common condition in diabetic patients. Susceptibility to periodontitis increased by around threefold in diabetic patients [35]. Evidence suggests that managing or preventing one could aid in alleviating the other condition. Thus, treating or preventing periodontitis may improve the status of other chronic conditions.

By belonging to a health group, a primary care physician can get instant expectations on patient’s health, and which preventive tests a patient might need. The multiple factors of a CD, for most physicians, can sometimes be rather time-consuming to collect; and so our ML model aids in providing comprehensive consideration of hundreds of variables. We aim to experiment with this model at small scale facilities first, such as at a university healthcare facility or a clinic. Further clustering data results and code are available for researchers upon request from the authors.

Validating unsupervised models is an intricate task. For the clustering model, we applied three main means of evaluation: (1) Relative clustering validation: evaluates the clustering model by varying different parameter values for the same algorithm. In this study, we evaluated a different number of clusters (k), however, as part of future work, we would like to test including/excluding other healthcare variables and observe the changes to the model outputs. (2) External clustering validation: compares the results of a cluster analysis to an externally known result, such as externally provided NHANES clusters (which are not provided by NHANES). Since we know the best cluster number in advance (k = 11 or k = 5), this approach is mainly used for selecting the right clustering algorithm. 3. Internal clustering validation: uses the internal workings of the clustering process without reference to external knowledge. This type aims to minimize the distance between data points in the same cluster and maximize ones in different clusters. Different diseases require different measures, and therefore, a breakdown of CD patients within every cluster is presented in Table 3 (*result #7*).

**Table 3:**
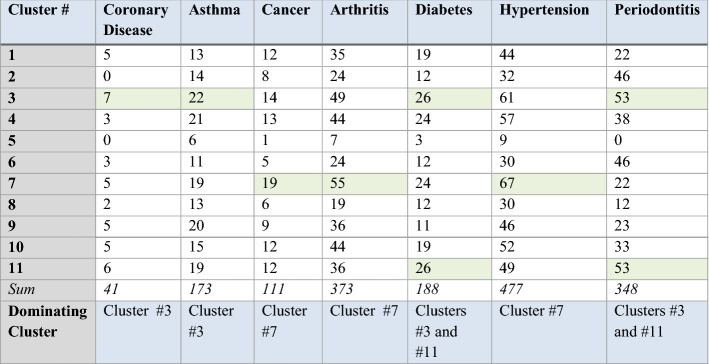
Chronic diseases’ distribution amongst clusters

As noted in the table, if a patient ends up in cluster # 3 for instance, it is safe to assume that they have health parameters that are very similar to a group of other patients with Coronary disease, or Asthma. Additionally, if a patient belongs to clusters #5 or #8, then their health parameters are similar to a group of healthy patients. The next section presents conclusions and implications on healthcare policy.

## Discussion: conclusions, policies, and future plans

Due to patient-privacy rules and legal concerns, accessible data in healthcare are scarce. Survey datasets from CDC/NHANES constitute an example datasets that could be used to provide pointers to the current general health of Americans. The surveys have been continuously used to draw causal inferences from health attributes, patient’s information, and diseases. NHANES datasets are complex, highly context-dependent, inherently heterogeneous, and multi-dimensional. Just like any half-cleaned dataset, data wrangling methods need to be applied before developing models that aid in decision making [36]. Besides manually evaluating patient records and health indicators by physicians, ML models presented in this manuscript allow *for a proactive evaluation of patients’ status*. ML models aim to augment physicians’ tasks, encourage preventive actions towards diseases, and provide pointers to healthcare policies that work (or others that do not)—a challenging task that requires systematic data democratization [37].

The methods presented in this paper provide successful imputations, correlations, and grouping of patients; as suggested in the hypothesis. We present seven analytical results. Ten years of NHANES data are collected and multiple insights are presented, for example: demographical data have ~ 40,000 patients (after joining with healthcare variables)—however, demographical categories are not balanced, for instance: the majority of surveyed patient’s household income is $100,000 and more. In any case, NHANES data have enough health indicators to further impute preventive measures. As we ran multiple imputation tests, dependable data are generated through a 6-step process with a very low error rate: 0.071. Imputations are deployed based on high correlations (77% and higher) of variables relevant to preventive measures (based on the ACA benchmark). Some presented columns had very high correlations (up to: 97.2%). Afterwards, data are clustered. Eleven (11) clusters served the highest quality and distribution of patients amongst heterogeneous groups. Smoking and periodontitis preventive measures are clearly stated. Other CDs (such as diabetes and asthma) were very prevalent amongst clusters 3, 7 and 11.

When a patient falls into a cluster, the physician can have an initial pointer to the health of the patient (in terms of what preventive tests to undertake) [38]. The overarching goal is to identify health and disease parameters for the US population, elevate the health of the American public, effectively reduce office time for patients’ data collection, and provide completed, wrangled, and unbiased survey datasets for further clinical analysis.

### Big Data Analytics for policy making in healthcare

The ACA mandate encourages citizens to benefit from a list of 21 preventive parameters (Appendix 1). As our work indicates, three factors (immunization, access, and chronic disease) affect the efforts of national preventive care. Changes to policies in American states through intelligent recommendations could be driven by imputation and clustering models. Five example policy making cases are presented next.

#### Case #1: healthcare fraud

Healthcare fraud is on the rise, recommendations to patients by clinics or pharmaceutical companies are frequently not supported by explainable scientific proof; rather, merely through experience or bureaucratic governmental approvals. Intelligent recommendations and predictions could provide detailed categorizations and validations to patients (i.e. precision medicine). Almost every year in the US, there is a case of statewide healthcare fraud. Many times, the story behind such activities is the overuse of tests and medical examinations, ‘fake’ lab results, or ‘fake’ (placebo) medicine. Recommendations by fraudulent physicians/clinics/labs are usually not supported by proof, rather, only by *opinion*. Combining medical knowledge and expertise with an interactive data system can provide intelligent recommendations and further validation to patients. Accordingly, policies for the validation of clinical recommendations ought to be enforced.

#### Case #2: medical deserts

Another case where ML-driven methods could be very beneficial to the health of a state is when they are applied across what is referred to as *medical deserts*. In the US, there are wide areas in the heartland where there is very low access to healthcare (few doctors or clinics)—an automated recommendation system can help in some cases, and cover some of these areas. Multiple clinical and subclinical tests are implemented to acquire comprehensive knowledge to managing diseases and preventing them or their progression. Patients’ stratification and classification could aid in intelligently choosing appropriate tests; thus, minimizing the need for unnecessary access, and lowering health costs for patients who live in such areas.

#### Case #3: preventive consumption

Methods presented in this manuscript provide pointers to *preventive* policies that benefit the general good of Americans. For example, pharmaceutical industries benefit from the lack of preventive practices, simply because that will allow them to sell more medication to patients that don’t undergo preventive care. Additionally, food and agricultural industries are able to push products to consumers that could cause chronic diseases (such as hypertension and diabetes). Diet for example, has major effects on health variables; however, medical institutions and policy makers are involved with cure but rarely with prevention. Our study evaluates the biomarkers concerned with diet and points to attributes that could lead to CDs.

#### Case #4: defensive medicine

Some clinical tests are expensive for patients and are time consuming for hospitals. ML methods can help in providing *cheaper* solutions. The models allow doctors to recommend preventive measures to patients based on what group they belong to instead of performing all measures to avoid being sued (less tests might be recommended if the patient belongs to cluster 5 for instance). Cluster 5 had only 26 patients with a CD; cluster 8 had 94, and cluster 6 had 131. On the other hand, cluster 3 (the least healthy cluster) had 232 CD patients, and cluster 7 had 211 patients. Therefore, in a clinical setup, a patient that belongs to cluster 5 will require less testing than a patient belonging to clusters 3 or 7. This will provide a mechanism to avoid over-recommending, or under-recommending clinical tests. Over-recommending tests is a case of *Defensive Medicine,* and as established prior, this and other practices have been on the forefront of reasons to the increasing expenditures within the US healthcare system.

#### Case #5: immunizations

Our ML methods can also aid in immunization recommendations and e-access to healthcare. For example, CDC can provide ML models for patients to self-evaluate, and quickly get predicted metrics to their immunization needs (with a certain statistical confidence—error rate in this study is *0.071*, and R-squared for all tested cases is higher than *70%*).

In the future, we aim to apply further experimentation on our system, such as: 1. Run models per US state: as different states have different rules and regulations, we aim to re-train models per state, and maybe also per county and city. 2. We aim to collect more CDC data variables to provide more correlations and further tests for imputations, and compare with other NHANES predictive models for specific diseases such as periodontitis [39]. 3. *K *= *5* and *k *= *7* models are to be tested on the same dataset to further evaluate their usability for medical purposes. 4. We aim to develop a user interface for clinicians to allow for interaction with ML models for decision making support.

The recommendations through our models are not a replacement for visiting a primary care physician or models like the Andersen model [40]; however, they are tools that could keep the citizens informed about their own health and would allow them to take preventive parameters (on time) if needed. When such big data analytics are applied to a state or to the national scale, they can provide recommendations to policies based on the aggregated health of citizens in a state. That will eventually promote the health of citizens across the country, something that is currently at the forefront of American policy making.

## Data Availability

The datasets generated and/or analyzed during the current study are available in the CDC/NHANES repository, https://www.cdc.gov/nchs/nhanes/index.htm All the datasets, the R code, and the SQL database are available to interested readers (via a GitHub repository) upon request from the authors.

## Appendix

**Table:**

Biomarker ID	ACA preventive measure name/description
1	Abdominal aortic aneurysm one-time screening for men of specified ages who have ever smoked
2	Alcohol misuse screening and counseling
3	Aspirin use to prevent cardiovascular disease and colorectal cancer for adults 50 to 59 years with a high cardiovascular risk
4	Blood pressure screening
5	Cholesterol screening for adults of certain ages or at higher risk
6	Colorectal cancer screening for adults 50 to 75
7	Depression screening
8	Diabetes (Type II) screening for adults 40 to 70 years who are overweight or obese
9	Diet counseling for adults at higher risk for chronic disease
10	Falls prevention (with exercise or physical therapy and vitamin D use) for adults 65 years and over, living in a community setting
11	Hepatitis B screening for people at high risk, including people from countries with 2% or more Hepatitis B prevalence, and US-born people not vaccinated as infants and with at least one parent born in a region with 8% or more Hepatitis B prevalence.
12	Hepatitis C screening for adults at increased risk, and one time for everyone born 1945–1965
13	HIV screening for everyone ages 15 to 65, and other ages at increased risk
14	Immunization vaccines for adults (such as Mumps, and Measles)
15	Lung cancer screening for adults 55–80 at high risk for lung cancer because they’re heavy smokers or have quit in the past 15 years
16	Obesity screening and counseling
17	Sexually transmitted infection (STI) prevention counseling for adults at higher risk
18	Statin preventive medication for adults 40 to 75 at high risk
19	Syphilis screening for adults at higher risk
20	Tobacco use screening for all adults and cessation interventions for tobacco users
21	Tuberculosis screening for certain adults without symptoms at high risk

## Abbreviations

ACA: Affordable Care Act
ML: Machine Learning
CDC: Centers for Disease Control and Prevention
IOM: Institute of Medicine
VPDs: Vaccine Preventable Diseases
NMEs: Non-Medical Exemptions
CD: Chronic disease
AR: Arkansas
AZ: Arizona
ID: Idaho
ME: Maine
MN: Minnesota
ND: North Dakota
OH: Ohio
OK: Oklahoma
OR: Oregon
PA: Pennsylvania
TX: Texas
UT: Utah
CDPP: Chronic Disease Prevention and Prediction
AI: Artificial Intelligence
KBS: Knowledge Based Systems
GPS: General problem solver
CERC: Center for Clinical Excellence
SMQ: Smoking
HIPPA: Health Information Privacy
